# An Interesting Clinical Case

**Published:** 1903-11

**Authors:** 


### Abstracts and Selections.

### An Interesting Clinical Case.

X, a white woman, 22 years of age, was taken into the hospital on account of syphilitic skin disease (roseola papula); a blennorrhagic gavinitis of most violent description, with strong congestion of the mucous membranes of the vagina. The latter was of violet hue, somewhat brittle, and yielded abundant secretion of a greenish-yellow pus, which showed under bacteriological examination abundant colonies typical of gonococcus, diplococcus and other varieties of bacteria. The gonococci infection reached to the neck of the uterus, whose tissues suffered from the same degeneration as the vagina. Above the mouth of the neck—from which a greenish-yellow and somewhat thick pus oozed—was a syphilitic ulcer of the size of a dime, clean at the bottom, livid in color and rather deep.

Upon careful examination, the patient was found to be pregnant in the third month; and, from the start, was subjected to energetic treatment as a serious case.

Under the treatment employed she improved rather well; but, though the blennorrhagia was not cured, the syphilitic manifestations of the skin disappeared, and the ulcer at the neck improved somewhat, until confinement, which took place at the eighth month, five months after her admission.

The confinement was normal. However, the patient was attacked by a great flux and suffered a complete laceration of the right side of the neck; an incomplete laceration of the left side; an incomplete laceration of the rear wall of the vagina; and a two-thirds laceration of the perineum. The placenta was removed at once; ample warm washes of a 1 per cent solution of permanganate of potash were applied and the uterus was stimulated by massage, but remained inert. All this was reported to me by the house physician. I arrived at the hospital four hours later in company with the well known gynecologist, Dr. Mendez Capote, who, upon having examined the patient, decided to sew up the lacerations. He washed out the vagina and uterine cavity completely; adjusted with the scissors the edges of the lacerated tissues; sewed up the wounds and touched the ulcer at the neck with the cauterizer; then he gave another wash and plugged with iodoform gauze.

When the patient was on the operating table, she had fever, 38.4° C. At 5 p. m. the fever was at 39°; then the vaginal plug was taken out and a great intra-uterine wash of one-half per cent. solution of permanganate was applied very hot in a quantity of five liters. The fever was at 40° throughout the night, and washes were given every four hours.

The following day, at 8 a. m., temperature 40°, same local treatment. The fever lasted all day, falling to 39° by the wash, but rose again to 40°.

The day thereafter, fever at 41°; same treatment with more vaginal washes of bichloride of mercury, before the uterine washes; the fever keeps on at 41°.

On the next day at 8 a. m. (temperature 41.5°), I took out the stitches made on the day of confinement, washed well both uterus and vagina, dried the latter with carbolated cotton and conveyed into the uterine cavity eight grammes of pure hydrozone, taking care that this liquid should flow towards the vagina, into which I poured about sixty grammes of the same liquid and drained the uterus with simple gauze saturated in hydrozone, while the vagina was drained by the same means.

From that time on the fever declined slowly, and at 6 p. m. it was apyretic. The fever did not return and the patient's cure proceeded without further difficulty.

This case, which is interesting by itself, proves of great value in setting forth two points, viz.:

1. That, although the intra-uterine injections of our hydrozone may be dangerous, it can be applied if care is taken to keep the neck dilated as much as possible.

2. That in this case the superiority of hydrozone over the other treatments of puerperal septicæmia, in connection with gonococci, is indisputable; and that this splendid result should encourage repetition.*—Dr. Matias Duque, Director of the San Antonio Hospital, Section of Hygiene. Abstract from the *Revista Medica Cubana,* April 15, 1903.

*The son of the patient suffered from blennorrhagia in the eyes. He was treated with one-four per cent solution of permanganate and installations of pure hydrozone twice daily, alterating with cauterizations of 40 per cent solution of nitrate of silver; and he kept his sight.

